# Expositionsanfragen zu Zubereitungen mit pflanzlichen Bestandteilen in Deutschland: Analyse von Anfragen (2013–2022) am Gemeinsamen Giftinformationszentrum Erfurt

**DOI:** 10.1007/s00103-026-04237-3

**Published:** 2026-04-29

**Authors:** Sebastian Wendt, Daniel Linden, Dagmar Prasa, Heike Franke

**Affiliations:** 1 Stabsstelle Krankenhaushygiene, Universitätsklinikum Halle (Saale), Magdeburger Str. 24, 06112 Halle (Saale), Deutschland; 2 Deutsches Beratungszentrum für Hygiene (BZH GmbH), Freiburg/Breisgau, Deutschland; 3 Gemeinsames Giftinformationszentrum der Länder Mecklenburg-Vorpommern, Sachsen, Sachsen-Anhalt und Thüringen, HELIOS Klinikum Erfurt, Erfurt, Deutschland; 4 Rudolf-Boehm-Institut für Pharmakologie und Toxikologie, Medizinische Fakultät, Universität Leipzig, Leipzig, Deutschland

**Keywords:** Pflanzliche Arzneimittel, Vergiftungen, Intoxikationen, Expositionen, Giftinformationszentrum, Herbal medicinal products, Poisoning, Intoxication, Exposure, Poison information center

## Abstract

**Hintergrund:**

Zubereitungen mit pflanzlichen Bestandteilen, wie sie in pflanzlichen Arzneimitteln, Medizinprodukten, Nahrungsergänzungsmitteln und Kosmetika enthalten sind, sind leicht zugänglich und können bei Anwendungsfehlern zu Nebenwirkungen oder Vergiftungen führen. Giftinformationszentren (GIZ) beantworten Expositionsanfragen. Ziel dieser Studie war es, Expositionsanfragen zu Zubereitungen mit pflanzlichen Bestandteilen am Gemeinsamen Giftinformationszentrum Erfurt (GGIZ) auszuwerten, um Präventionsansätze zu identifizieren.

**Methoden:**

Es wurde eine statistische Auswertung der Expositionsanfragen am GGIZ für 2013–2022 durchgeführt. Daten aus 9 Bundesländern flossen ein. Berücksichtigt wurden Anfragen zu Einzelpräparaten, die mindestens 10-mal angefragt wurden. Die Symptomschwere wurde mit dem Poisoning Severity Score (PSS) und das Vergiftungsrisiko mit einem modifizierten Litovitz-Risikofaktor berechnet.

**Ergebnisse:**

36 Zubereitungen mit 2512 Expositionen (Ø251,2/a; Trend τ = 0,8667, *p* = 0,0007) wurden identifiziert. 60,2 % (1511) der Betroffenen waren Kleinkinder. 98,5 % (2474) der Expositionen ereigneten sich im häuslichen Umfeld. In 64,5 % (1620) geschahen die Expositionen unabsichtlich. 93,4 % (2345) waren oral. 75,3 % (1780/2365) der Expositionen blieben symptomlos, 23,5 % (556/2365) hatten leichte/geringe, 1 % (24) mittlere/mäßige und 0,2 % (5) schwere Symptome. Schwere Intoxikationen traten durch Colchysat® Bürger und Klosterfrau® Melissengeist Konzentrat auf.

**Diskussion:**

Kleinkinder sind eine relevante Risikogruppe bei der Anwendung von Zubereitungen mit pflanzlichen Bestandteilen. Präparate mit Colchicin oder Ethanol können insbesondere bei intentionalen Expositionen zu tödlichen Intoxikationen führen. Es wird empfohlen, orale und extraorale Präparate getrennt und außerhalb der Reichweite Unbefugter zu lagern und umfassende Aufklärung anzubieten.

**Zusatzmaterial online:**

Zusätzliche Informationen sind in der Online-Version dieses Artikels (10.1007/s00103-026-04237-3) enthalten.

## Abstract (en)

**Background:**

Preparations containing plant-derived components, such as those found in herbal medicinal products, medical devices, dietary supplements, and cosmetics, are readily accessible and may lead to adverse effects or poisoning if used incorrectly. Poison information centers (PICs) respond to exposure-related inquiries. The aim of this study was to analyze exposure inquiries concerning preparations with plant-derived components at the Joint Poison Information Center in Erfurt (GGIZ) in order to identify preventive approaches.

**Methods:**

A statistical analysis of exposure inquiries received by the GGIZ from 2013 to 2022 was conducted. Data from nine federal states were included. Only inquiries related to single preparations that had been reported at least 10 times were considered. Symptom severity was assessed using the poisoning severity score (PSS), and poisoning risk was calculated using a modified Litovitz risk factor.

**Results:**

A total of 36 preparations with 2512 exposures (mean 251.2/year; trend τ = 0.8667, *p* = 0.0007) were identified. Of those affected, 60.2% (1511) were toddlers. Most exposures (98.5%, 2474) occurred in the home environment. In 64.5% (1620) of cases, exposures were unintentional. Oral exposure accounted for 93.4% (2345) of cases. Of the exposures with known outcomes, 75.3% (1780/2365) remained asymptomatic, 23.5% (556/2365) resulted in minor/mild symptoms, 1% (24) in moderate symptoms, and 0.2% (5) in severe symptoms. Severe intoxications were associated with Colchysat® Bürger and Klosterfrau® Melissengeist concentrate.

**Discussion:**

Toddlers represent a relevant risk group in the use of preparations containing plant-derived components. Preparations containing colchicine or ethanol may lead to fatal intoxications, particularly in cases of intentional exposure. It is recommended to store oral and topical preparations separately and out of reach of unauthorized persons, and to provide comprehensive education.

## Hintergrund

Anfragen zu Arzneimittelvergiftungen machen derzeit den größten Anteil aller Anfragen in den Giftinformationszentren aus. In den Jahren 2015 bis 2024 waren Arzneimittel im Einzugsbereich des Gemeinsamen Giftinformationszentrums in Erfurt (GGIZ) in 61 % der Vergiftungs- bzw. Vergiftungsverdachtsfälle bei Erwachsenen und in 33 % bei Kindern und Jugendlichen beteiligt [1].

Insbesondere pflanzliche Arzneimittel kommen bei vielen Erkrankungen wie „Erkältungskrankheiten“ häufig zum Einsatz (ca. 100–120 Mio. Packungen pro Jahr in Deutschland [2–4]). Viele Zubereitungen mit pflanzlichen Bestandteilen sind in Deutschland in Apotheken, Drogerien und sogar Supermärkten erhältlich – und somit für große Bevölkerungsteile zugänglich. Dennoch werden die Risiken und Nebenwirkungen oft aufgrund ihrer vermeintlichen „Natürlichkeit“ unterschätzt [2, 5]. Fallberichte, Reviews und Studien hingegen weisen auf toxikologische Risiken hin [3, 6–13]. Bis heute ist das Risikopotenzial von verschiedenen Zubereitungen mit pflanzlichen Bestandteilen noch nicht vollständig bekannt.

Ziel dieser Studie war es, Expositionsanfragen am GGIZ zu Zubereitungen mit pflanzlichen Bestandteilen in Form von Einzelpräparaten, einschließlich Symptomschwere und Intoxikationsrisiko, auszuwerten, um Präventionsansätze zu identifizieren. Erste Zwischenergebnisse wurden vorab als Kurzmitteilung („Häufigkeit von Anfragen zu befürchteten Intoxikationen mit pflanzenbasierten Arzneipräparaten“) im *Deutschen Ärzteblatt* publiziert [14]. Die vorliegende Vollpublikation liefert eine vollständige Datenauswertung mit detaillierten Subgruppenanalysen sowie eine vertiefte Darstellung schwerer und letaler Verläufe.

## Methoden

In dieser Arbeit wird der Begriff „Zubereitungen mit pflanzlichen Bestandteilen“ (kurz: Zubereitungen, Produkte, Präparate) als neutraler Oberbegriff für alle im Datensatz erfassten Produkte verwendet, die pflanzliche Ausgangsstoffe, pflanzliche Extrakte oder pflanzliche Vielstoffgemische enthalten. Das schließt pflanzliche Arzneimittel, traditionelle pflanzliche Arzneimittel (pflanzliche Arzneimittel mit langjähriger Anwendung ohne geforderten klinischen Wirksamkeitsnachweis), Medizinprodukte, Nahrungsergänzungsmittel sowie kosmetische Produkte ein, sofern diese relevante pflanzliche Inhaltsstoffe aufweisen.

Für die Expositionsbewertungen wurde eine Analyse von Anfragen am GGIZ Erfurt für 2013–2022 durchgeführt. Grundlage sind die GGIZ-Protokolldaten aus den Bundesländern Mecklenburg-Vorpommern, Sachsen, Sachsen-Anhalt und Thüringen sowie aus 5 weiteren Bundesländern im Rahmen einer Nachtdienstkooperation (Bremen, Hamburg, Niedersachsen, Schleswig-Holstein, Baden-Württemberg).

Eingeschlossen wurden Anfragen zu akuten und chronischen humanen Expositionen, die nach Einschätzung des GGIZ Erfurt mit großer Wahrscheinlichkeit auf ein bestimmtes Einzelpräparat (Monoexposition) zurückzuführen waren (Hauptnoxen- bzw. Kausalitätsbetrachtung). Um möglichst aussagekräftige Ergebnisse (u. a. zu Altersverteilung, Symptomschwere, medizinischem Interventionsbedarf) zu erhalten, wurden zunächst häufige („relevante“) Präparate mit einer festgesetzten Expositionsrate von *n* ≥ 10 im Studienzeitraum betrachtet (= 1 Anfrage/a).

Weiterführende Analysen (z. B. zu schweren Fällen, Todesfällen, Suiziden, ausgewählten Einzelpräparaten) beziehen sich auf den Gesamtdatensatz mit Anfrageraten von *n* ≥ 1 im Studienzeitraum.

Da im GGIZ nicht in allen Fällen vollständige Angaben zu sämtlichen Variablen vorliegen, kann sich bei der Ergebnisdarstellung die Gesamtzahl der ausgewerteten Fälle je nach analysiertem Merkmal unterscheiden.

Zur Beurteilung der Symptomschwere wurde der *Poisoning Severity Score* (PSS) benutzt ([15]; siehe *Onlinematerial *mit Kriterien des PSS zur klinischen Beurteilung der Vergiftungsschwere am GGIZ).

Altersgruppen werden standardmäßig nach GGIZ-Vorgaben wie folgt definiert: Baby (< 1 Jahr), Kleinkind (1 bis < 6 Jahre), Kind bzw. Jugendlicher (6 bis < 18 Jahre) und Erwachsener (≥ 18 Jahre).

Durch Berechnung eines modifizierten Litovitz-Risikofaktors konnten Präparate mit dem höchsten Vergiftungsrisiko identifiziert werden. Dazu wurden schwere und mittelschwere Intoxikationen addiert und auf 100 Anfragen zu einem Präparat bezogen. Anfragetrends wurden mittels Mann-Kendall-𝜏-Tendenztests (Signifikanzniveau α = 0,05) analysiert. Die statistische Auswertung und grafische Darstellung erfolgten mit MS Excel (Version 16.0, Redmond, USA) bzw. IBM SPSS (Version 24.0, Armonk, USA; Mann-Kendall-𝜏-Tendenztest). Zwischenergebnisse wurden vorab publiziert [14].

## Ergebnisse

### Globalanalyse

**Abb. 1:**
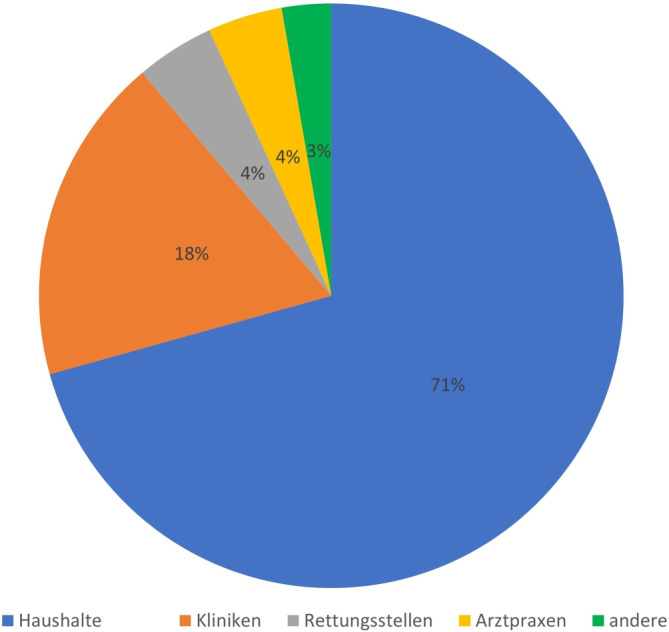
Herkunft aller Expositionsanfragen zu Zubereitungen mit pflanzlichen Bestandteilen am GGIZ im Studienzeitraum 2013–2022 (*n* = 3341)

Von insgesamt 197.815 angefragten Expositionen im Zeitraum 2013–2022 am GGIZ bezogen sich 100.535 (50,8 %) auf Arzneimittel. Der Anteil von Anfragen zu Präparaten mit pflanzlichen Bestandteilen betrug 3,3 % (3341/100.535 bei Hauptnoxenbetrachtung). Ein Großteil dieser Expositionsanfragen ging auf private Haushalte (70,7 %; 2361) zurück, ein kleinerer Anteil auf Kliniken (18,1 %; 606), Rettungsstellen (4,3 %; 145), Arztpraxen (4,1 %; 138) oder andere Institutionen (2,7 %; 91, u. a. Notdienste der Kassenärztlichen Vereinigungen, Apotheken, Heime/Wohngruppen, Kindergärten/Schulen, Pflegedienste; Abb. 1).

**Abb. 2:**
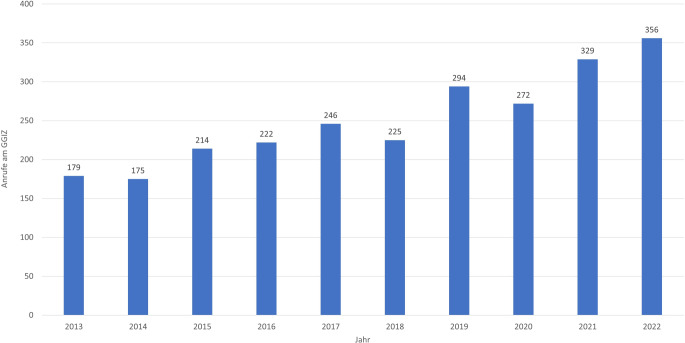
Expositionsanfragen für Zubereitungen mit pflanzlichen Bestandteilen im Studienzeitraum 2013–2022 am GGIZ – ausgewertet für Produkte mit mindestens 10 Anfragen im Untersuchungszeitraum (*n* = 2512)

Insgesamt betrafen die Expositionen 366 verschiedene Präparate. Bei einer Anfragerate von *n* ≥ 10 und der Betrachtung von Einzelpräparaten verblieben 36 Präparate mit 2512 Expositionen für die weiterführende Analyse (Tab. 1). Die diesbezüglichen Expositionsraten (Anfrageraten) schwankten zwischen 175 im Jahr 2014 und 356 im Jahr 2022 mit durchschnittlich 251 Anfragen pro Jahr und einem insgesamt steigenden Trend über die Studienjahre (𝜏 = 0,8667, *p* = 0,0007, Abb. 2). Es gab keinen signifikanten Unterschied bezüglich der Geschlechterverteilung (männlich versus weiblich; Zeitraum 2013–2022; Chi^2^ = 0,769, *p* = 0,3804).

**Tab. 1:** Expositionsanfragen zu Produkten, die Zubereitungen mit pflanzlichen Bestandteilen enthalten, am GGIZ Erfurt im Zeitraum 2013–2022, sortiert nach Häufigkeit (*n* = 2512), einschließlich Symptomschweregrade und Vergiftungsrisiko (modifizierter Litovitz-Risikofaktor). Enthalten sind Produkte, für die im Zeitraum mindestens 10 Anfragen eingingen

Ranking-Nummer	Präparat	Wirkstoffkomponenten	Hauptanwendung (Applikationsform)	Aufnahmeweg nach Fachinformation	Toxikologisch relevante Aufnahmewege am GGIZ	Expositionen (2013–2022)	Schweregrad der Symptome					Modifizierter Litovitz-Risikofaktor
							Symptomlos	Leicht	Mäßig	Schwer (inkl. Tod)	*Nicht einschätzbar oder unbekannt*	
**1**	Prospan® Hustensaft, Engelhard Arzneimittel GmbH & Co. KG, Niederdorfelden, Deutschland	Efeublätter-Trockenextrakt	Husten (oral)	Oral	Oral, okulär	**562**	433	105	3	0	21	0,5
**2**	Babix®-Inhalat N, Heinrich Mickan Arzneimittel GmbH & Co. KG, Karlsruhe, Deutschland	Eukalyptusöl, Fichtennadelöl	Erkältung (inhalativ)	Inhalativ	Oral, dermal, okulär, inhalativ, i.v.	**439**	348	71	0	0	20	0
**3**	*Minzöl*	Minzöl	Erkältung (inhalativ)	Inhalativ, oral, dermal	Oral, dermal, okulär, inhalativ	**194**	94	82	3	0	15	1,5
**4**	Sanopin® wern (Eucabal® Inhalat), Aristo Pharma GmbH, Berlin, Deutschland	Eukalyptusöl, Kiefernnadelöl	Erkältung (inhalativ)	Inhalativ	Oral	**158**	114	31	1	0	12	0,6
**5**	*Erkältungsbad*	Verschiedene ätherische Öle	Erkältung (inhalativ)	Inhalativ	Oral, inhalativ, okulär, dermal	**93**	58	28	2	0	5	2,2
**6**	Eucabal®-Balsam S, Aristo Pharma GmbH, Berlin, Deutschland	Eukalyptusöl, Kiefernnadelöl	Erkältung (dermal/inhalativ)	Dermal-inhalativ (Creme)	Oral, inhalativ, okulär, dermal	**88**	71	10	1	0	6	1,1
**7**	Bronchipret® Saft TE, Bionorica SE, Neumarkt, Deutschland	Thymianfluidextrakt, Efeublätterfluidextrakt	Husten (oral)	Oral	Oral	**87**	76	3	0	0	8	0
**8**	*Lavendelöl*	Lavendelöl	Unruhe (dermal/inhalativ)	Oral, dermal-inhalativ	Oral, dermal, inhalativ	**83**	63	14	0	0	6	0
**9**	*Kümmelöl*	Kümmelöl	Blähungen (dermal/oral)	Oral, dermal-inhalativ	Dermal, oral	**75**	59	14	0	0	2	0
**10**	*Fenchel-Kümmel-Öl*	Fenchelöl, Kümmelöl	Blähungen (dermal/oral)	Oral	Oral, dermal	**68**	61	5	0	0	2	0
**11**	*Eukalyptusöl*	Eukalyptusöl	Erkältung (inhalativ)	Oral, dermal-inhalativ	Oral, inhalativ, dermal, okulär	**61**	30	28	1	0	2	1,6
**12**	Prospan® Hustenliquid, Engelhard Arzneimittel GmbH & Co. KG, Niederdorfelden, Deutschland	Efeublätter-Trockenextrakt	Husten (oral)	Oral	Oral, okulär	**60**	43	14	0	0	3	0
**13**	*Ätherisches Öl*	Verschiedene ätherische Öle	u. a. Erkältung (dermal/inhalativ)	Oral, inhalativ	Oral, inhalativ, nasal, dermal	**53**	35	13	1	0	4	1,9
**14**	Wick® VapoRub Erkältungssalbe, WICK Pharma, Schwalbach am Taunus, Deutschland	Levomenthol, racemischer Campher, Eukalyptusöl, gereinigtes Terpentinöl	Erkältung (dermal/inhalativ)	Dermal-inhalativ	Oral, dermal, inhalativ	**51**	44	7	0	0	0	0
**15**	*Franzbranntwein*	u. a. natürlicher Campher (D- und DL-Form), Levomenthol	Muskelbeschwerden (dermal)	Oral, dermal	Oral, nasal, dermal	**49**	24	12	3	0	10	6,1
**16**	*Baldrianwurzel*	Baldrianwurzel-Auszüge	Unruhe, Schlafstörungen (oral)	Oral	Oral	**46**	27	12	0	0	7	0
**17**	*Nelkenöl*	Gewürznelkenöl	Zahnschmerzen (lokal/oral)	Oral, inhalativ	Oral, dermal	**39**	21	18	0	0	0	0
**18**	Colchysat® Bürger^a^ Johannes Bürger Ysatfabrik GmbH, Bad Harzburg, Deutschland	Herbstzeitlosenblüten-Extrakt	Gicht (oral)	Oral	Oral	**29**	5	13	5	4^b^	2	31
**19**	*Erkältungsbalsam*	Verschiedene ätherische Öle	Erkältung (dermal/inhalativ)	Dermal-inhalativ	Oral, dermal, inhalativ, okulär	**24**	13	7	1	0	3	4,2
**20**	Pulmotin® (Erkältungstropfen, Salbe), Serumwerk Bernburg AG, Bernburg, Deutschland	Eukalyptusöl, Sternanisöl, Thymol, racemischer Campher, Coniferennadelöle, Thymianöl vom Thymol-Typ	Erkältung (dermal/oral/inhalativ)	Dermal-inhalativ bzw. oral (Tropfen)	Oral, dermal, inhalativ	**24**	18	3	2	0	1	8,3
**21**	Phytohustil® Hustenreizstiller, Bayer Vital GmbH, Leverkusen, Deutschland	Eibischwurzel-Extrakt	Reizhusten (oral)	Oral	Oral	**20**	19	1	0	0	0	0
**22**	Umckaloabo® Saft für Kinder, Dr. Willmar Schwabe GmbH & Co. KG, Karlsruhe, Deutschland	*Pelargonium-sidoides*-Wurzel-Trockenextrakt	Akute Bronchitis (oral)	Oral	Oral	**19**	18	0	0	0	1	0
**23**	Iberogast®, Bayer Vital GmbH, Leverkusen, Deutschland	Auszüge von Schleifenblumen, Angelikawurzel, Kamillenblüten, Kümmel, Mariendistelfrüchte, Melissenblätter, Pfefferminzblätter, Schöllkraut, Süßholzwurzel	Funktionelle Dyspepsie (oral)	Oral	Oral	**18**	12	3	1	0	2	5,6
**24**	*Thymianöl*	Thymianöl	Erkältung (inhalativ)	Oral	Oral, dermal, okulär, inhalativ	**17**	5	10	0	0	2	0
**25**	*Erkältungsöl*	Verschiedene ätherische Öle	Erkältung (dermal/inhalativ)	Oral, inhalativ	Oral, inhalativ	**16**	12	4	0	0	0	0
**26**	*Fenchelöl*	Fenchelöl	Blähungen (oral/dermal)	Oral	Oral	**16**	14	2	0	0	0	0
**27**	GeloMyrtol® forte, G. Pohl-Boskamp GmbH & Co. KG, Hohenlockstedt, Deutschland	Eukalyptus‑, Süßorangen‑, Myrthen‑, Zitronenöl	Bronchitis, Sinusitis (oral)	Oral	Oral	**15**	9	4	0	0	2	0
**28**	Goldgeist® forte, Eduard Gerlach GmbH, Lübbecke, Deutschland	Pyrethrumblüten-Extrakt	Läusebefall (dermal)	Dermal	Oral, dermal, okulär	**15**	6	7	0	0	2	0
**29**	Transpulmin® Erkältungsbalsam für Kinder, Cooper Consumer Health Deutschland GmbH, Mannheim, Deutschland	Eukalyptusöl, Kiefernnadelöl	Erkältung (dermal/inhalativ)	Dermal-inhalativ	Oral, dermal	**15**	13	2	0	0	0	0
**30**	*Pfefferminzöl*	Pfefferminzöl	Erkältung (inhalativ)	Oral	Oral, dermal, okulär	**13**	9	4	0	0	0	0
**31**	*Johanniskraut*	Johanniskrautauszüge	Depressive Verstimmung (oral)	Oral	Oral	**12**	5	4	0	0	3	0
**32**	Klosterfrau® Melissengeist Konzentrat, MCM Klosterfrau Vertriebsgesellschaft mbH, Köln, Deutschland	Mischdestillat aus Melissenblätter, Alantwurzelstock, Angelikawurzel, Ingwerwurzelstock, Gewürznelken, Galgantwurzelstock, Schwarzer Pfeffer, Enzianwurzel, Muskatsamen, Bitterorangenschalen, Zimtrinde u. -blüten, Kardamom	Nervosität (oral)	Oral, dermal	Oral	**12**	2	6	0	1^c^	3	8,3
**33**	*Salbei-Tee*	Salbeiblätterauszüge	Halsschmerzen (oral/gurgeln)	Oral	Oral	**11**	2	8	0	0	1	0
**34**	*Baldrian-Tropfen*	Baldrianwurzelauszüge	Unruhe, Schlafstörungen (oral)	Oral	Oral	**10**	4	4	0	0	2	0
**35**	Salviathymol® N Madaus, Cooper Consumer Health Deutschland GmbH, Mannheim	Salbei‑, Eukalyptus‑, Pfefferminz‑, Zimt‑, Nelken‑, Bitterfenchel‑, Sternanisöl, Levomenthol, Thymol	Mundschleimhautentzündung (lokal)	Oral	Oral	**10**	6	4	0	0	0	0
**36**	Tetesept® Erkältungszeit Bad, Merz Lifecare/Merz Group, Frankfurt am Main, Deutschland	Eukalyptus‑, Terpentin‑, Thymian‑, Kiefernnadelöl, racemischer Campher	Erkältung (Bad/inhalativ)	Dermal-inhalativ	Oral, dermal okulär, inhalativ	**10**	7	3	0	0	0	0

() = Nachfolgeprodukte. Kursivschrift = Zusammenfassungen verschiedener Produkte mit identischen oder vergleichbaren Inhaltsstoffen. Modifizierter Litovitz-Risikofaktor: Anzahl der mittelschweren und schweren Symptome pro 100 Expositionen. a: Verschreibungspflichtig. b: Enthält einen nichtsuizidalen Todesfall. c: Die hier beschriebene Symptomatik aus „Koma mit Azidose“ sowie die Ingestionsmenge von 1,5 L sind Indizien für eine Ethanolintoxikation als Todesursache.

Die meisten Expositionsanfragen am GGIZ Erfurt kamen aus Sachsen (33,9 %; 851), gefolgt von Thüringen (15,2 %; 381), Sachsen-Anhalt (10,0 %; 250) und Mecklenburg-Vorpommern (8,0 %; 201). Mit Abstand am häufigsten war der Expositionsort das häusliche Umfeld (98,5 %; 2474). Zu 93,4 % (2345) handelt es sich um orale Expositionen.

Die 5 häufigsten Anfragen bezogen sich auf die Einzelpräparate Prospan® Hustensaft (22,4 %; 562/2512), Babix®-Inhalat N (17,5 %; 439/2512), Minzöl (*verschiedene Hersteller;* 7,7 %; 194/2512), Sanopin® wern Inhalat (6,3 %; 158/2512) und Erkältungsbad (*verschiedene Hersteller;* 3,7 %; 93/2512; Tab. 1). Abb. 5 zeigt ausgewählte Heilpflanzen und -bestandteile, die Teil der Zubereitungen verschiedener Präparate sind.

**Abb. 3:**
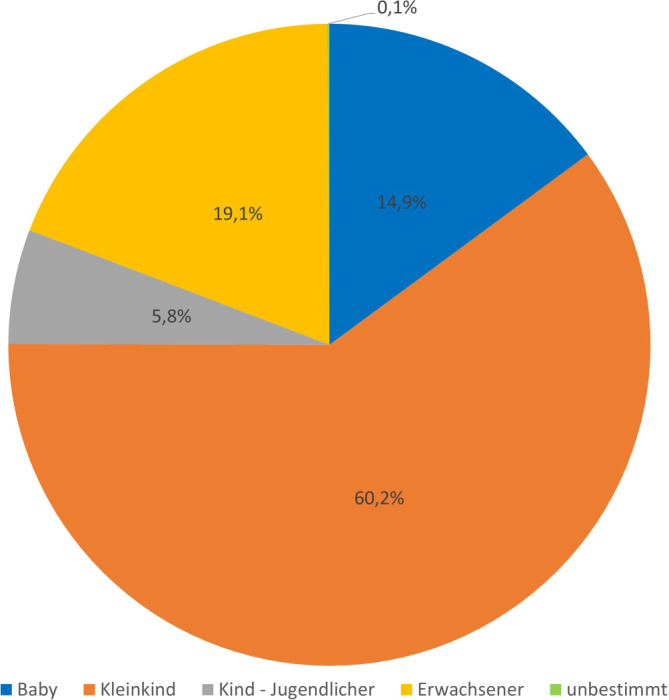
Altersverteilung bei den Expositionsanfragen zu Zubereitungen mit pflanzlichen Bestandteilen am GGIZ Erfurt im Zeitraum 2013–2022 (*n* = 2512)

**Abb. 4:**
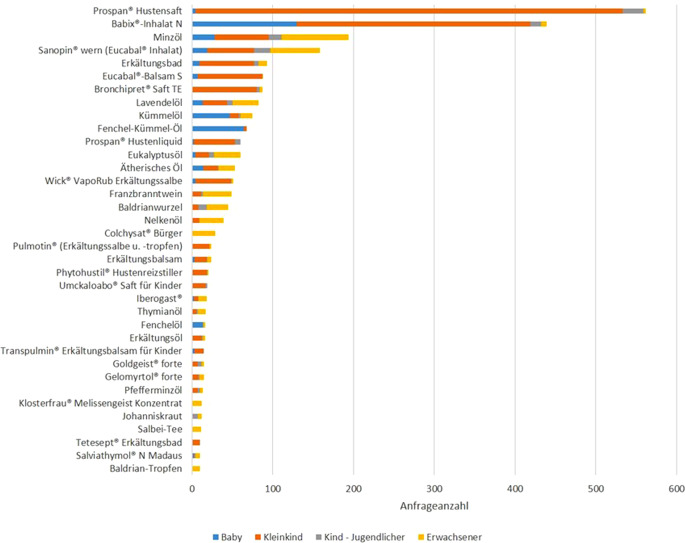
Aufschlüsselung der Altersverteilung aus Abb. 3 nach Einzelpräparaten

Die Altersstruktur setzte sich wie folgt zusammen: 60,2 % (*n* = 1511) waren Kleinkinder, 19,1 % (*n* = 479) Erwachsene, 14,9 % (*n* = 374) Babys, 5,8 % (*n* = 145) Kinder bzw. Jugendliche und 0,1 % (*n* = 3) mit unbekanntem Alter (Abb. 3). Kleinkinder waren auch bei Expositionen mit den beiden insgesamt am häufigsten vertretenen Präparaten Prospan® Hustensaft und Babix®-Inhalat N mit 94,1 % (529/562) bzw. 65,8 % (289/439) involviert (Abb. 4). Bei Kümmel- (62,7 %; 47/75) bzw. Fenchel-Kümmel-Öl (*je verschiedene Hersteller*; 94,1 %; 64/68) gab es den überwiegenden Beratungsbedarf für die Gruppe der Babys. Erwachsene waren – bei insgesamt geringeren Expositionsraten – bezüglich der Präparate Klosterfrau® Melissengeist Konzentrat (100 %; 12/12), Colchysat® Bürger (96,6 %; 28/29, verschreibungspflichtig!), Salbei-Tee (90,9 %; 10/11), Baldrian-Tropfen (90,0 %; 9/10), Franzbranntwein (73,5 %; 36/49), Nelken- (76,9 %; 30/39) und Thymianöl (*je verschiedene Hersteller;* 58,8; 10/17) überproportional häufig beteiligt.

Die Expositionsursachen waren zum größten Teil unabsichtlich (akzidentell; 64,5 %; 1620 – exklusive Verpackungsverwechslungen) oder begründen sich durch Präparateverwechslungen (25,0 %; 629); seltener war die Ursache ein unsachgemäßer Gebrauch (4,2 %; 106). Im Rahmen von Suizidversuchen wurden Zubereitungen mit pflanzlichen Bestandteilen sehr selten eingesetzt (1,8 %; 46): u. a. in 29 Fällen Medikamente mit Baldrian.

Ein unsachgemäßer Gebrauch für intendierte psychoaktive Zwecke war sehr selten (0,6 %; 14): Franzbranntwein (*n* = 6), Klosterfrau® Melissengeist Konzentrat (*n* = 3), Baldrian-Tropfen (*n* = 2), Lavendelöl (*n* = 1), Baldrianwurzel-Zubereitungen (*n* = 1) und ein nicht näher spezifiziertes „Erkältungsbad“ (*n* = 1).

75,3 % (1780/2365) aller bewerteten Expositionen blieben symptomlos; 23,5 % (556/2365) der Expositionsfolgen wurden als leicht/gering, 1,0 % (24/2365) als mittel/mäßig und 0,2 % (5/2365) als schwer eingestuft; bei 147 von insgesamt 2512 Expositionen war der Schweregrad nicht klar einschätzbar (in der %‑Rechnung nicht berücksichtigt). Die 5 schweren Expositionsfolgen mit relevanten Präparaten wurden durch Colchysat® Bürger (4 Erwachsene, orale Gesamtdosen ca. 22,5–50 mg Colchicin) und Klosterfrau® Melissengeist Konzentrat (1 Erwachsener, orale Gesamtdosen ca. 620–930 g Ethanol) verursacht. Bei Betrachtung *aller* schweren Vergiftungsfolgen bezüglich Monointoxikationen (*n* = 3193, Anfragehäufigkeit *n* ≥ 1) konnten 2 weitere schwere Fälle mit folgenden Präparaten identifiziert werden: Colchicum-Dispert® (1 Erwachsener, orale Gesamtdosis ca. 25 mg Colchicin) sowie Bärlappkraut-Tee (*Lycopodii herba, *1 Erwachsener, Gesamtdosis: „2 Tassen Tee“).

Bei den 46 suizidalen Intoxikationen mit relevanten Zubereitungen (32 Erwachsene, 14 Minderjährige) wurden in 29 Fällen Baldrianpräparate, in 7 Fällen Johanniskrautpräparate, in 4 Fällen Colchysat® Bürger (inkl. 2 vollendete Suizidversuche bei Männern, Colchicin-Gesamtdosen: ca. 50 mg), in 3 Fällen Klosterfrau® Melissengeist Konzentrat (inkl. 1 vollendeter Suizidversuch bei einer Frau; Ethanol-Gesamtdosen: ca. 623–935 g), in 2 Fällen Franzbranntwein (Ethanol-Gesamtdosen: ca. 24–284 g) und in einem Fall Prospan® Hustensaft (*unbekannte Dosis*) verwendet. Insgesamt gab es somit bei den relevanten Präparaten 3 suizidale Todesfälle sowie zusätzlich einen Sterbefall aufgrund eines unsachgemäßen Dosisgebrauchs bei Colchysat® Bürger (Colchicin-Gesamtdosis: ca. 50 mg) bei einem Erwachsenen. Bei Betrachtung *aller* Expositionen (*n* = 3193, Expositionshäufigkeit *n* ≥ 1) konnte ferner noch ein suizidaler Todesfall einer erwachsenen Frau durch Colchicum-Dispert® (Colchicin-Gesamtdosis: ca. 25 mg) identifiziert werden (produktspezifische Gesamtanfragerate bei Monointoxikation über den gesamten Studienzeitraum: *n* = 6).

Das Symptomenspektrum bei den  Todesfällen durch colchicinhaltige Präparate bestand aus „diffusen Bauchschmerzen“, „rezidivierendem Erbrechen“, „blutigem Durchfall“, „akutem Nierenversagen“, „Lungenödem“ sowie einem laborchemischen Anstieg von kardialem Troponin, der Transaminasen sowie der Laktatdehydrogenase. Bei der tödlichen Vergiftung durch Klosterfrau® Melissengeist Konzentrat kam es zum intubationspflichtigen Koma bei alkoholischer Ketoazidose. Insgesamt gab es somit – bei Betrachtung des Gesamtdatensatzes – 5 Todesfälle.

Eine ärztliche oder Laienintervention wurde in 87,9 % (*n* = 2209) der Fälle mit relevanten Präparaten empfohlen, wobei eine stationäre Behandlung bei 5,9 % (*n* = 149) bzw. eine ambulante Intervention bei 8,2 % (*n* = 207) aller Exponierten angeraten wurde. Bei den stationären Behandlungsempfehlungen waren, bezogen auf die Einzeleinnahmen der Präparate, mit größeren Anteilen Colchysat® Bürger (72,4 %; 21/29), Klosterfrau® Melissengeist Konzentrat (66,7 %; 8/12), Baldrian-Tropfen (50 %; 5/10), Goldgeist® forte (46,7 %; 7/15), Baldrianwurzeln (43,5 %; 20/46), Johanniskraut (33,3 %; 4/12) sowie Franzbranntwein (32,7 % 16/49) vertreten.

Spezifische Einzelsymptome lassen sich aufgrund der großen Symptomenvielfalt in der Dokumentation sowie aufgrund relativ geringer Fallzahlen pro Präparat nicht sinnvoll auswerten. Die Symptome bei oralen Expositionen haben aber ihren Schwerpunkt im „gastrointestinalen Bereich“, z. B. Übelkeit, Bauchschmerzen, Erbrechen und Diarrhö.

Erwähnenswert ist bei 40 inhalativen Expositionen (*n* = 10 Babix®-Inhalat, *n* = 6 Erkältungsbad, *n* = 3 Erkältungsbalsam, *n* = 7 „Erkältungsöl“ bzw. „ätherisches Öl“, *n* = 2 Eucabal® Balsam S, *n* = 1 Eukalyptus-, *n* = 3 Lavendel-, *n* = 6 Minzöl, *n* = 1 Thymianöl sowie *n* = 1 Tetesept® Erkältungszeit Bad) das Symptomspektrum von „Halskratzen“, Störung des Geschmackssinns (Dyspepsie), Reizhusten, Müdigkeit, Schwindel (Vertigo), Kopfschmerzen (Cephalgien), Hyperaktivität, Erbrechen (Emesis) sowie akuten asthmatischen Beschwerden.

### Kleinkinder (1 bis <6 Jahre)

Aufgrund der relativ hohen Gesamtfallzahl bei Kleinkindern erfolgte eine gesonderte Betrachtung ausgewählter Parameter für diese Altersgruppe:

33 Einzelpräparate mit 1511 Anfragen mit insgesamt steigendem Anfragetrend (⌀151,1 Fälle/a, Range 106 bis 226 Fälle/a, 𝜏 = 0,8222, *p* = 0,0013) bzw. 20 relevante Einzelpräparate (*n* ≥ 10 Anfragen im Studienzeitraum) mit 1446 Expositionen spielten hier eine Rolle.

Im Ranking auf Wirkstoffebene sind u. a. Zubereitungen mit Efeublätterextrakten (38,4 %; 580/1511), Pfefferminzöl (4,9 %; 74/1511), Eibischwurzel (1,1 %; 17/1511), Baldrianwurzel (0,5 %; 8/1511) und Pyrethrum (0,4 %; 6/1511) vertreten. In Einzelfällen spielten Colchicin und Johanniskraut eine Rolle (je 0,1 %; 1/1511). Die 5 häufigsten Expositionsanfragen gab es zu den Einzelpräparaten Prospan® Hustensaft (35,0 %; 529/1511), Babix®-Inhalat N (19,1 %; 289/1511), Bronchipret® Saft TE und Eucabal® Balsam S (5,3 %; 80/1511) sowie verschiedenen Erkältungsbädern (4,5 %; 68/1511; exklusive Tetesept® Erkältungszeit Bad; Abb. 4). Die Expositionsursachen sind zum größten Teil akzidentell (88,5 %; 1337, Präparateverwechslungen separat erfasst) oder gehen auf Präparateverwechslungen (8,9 %; 135) durch Anwender zurück; seltener ist die Ursache ein unsachgemäßer Gebrauch (1,5 %; 22).

In 80,6 % (1164) der Expositionen, bei denen Angaben zu Symptomen bzw. Symptomfreiheit vorlagen (1445), traten keine klinischen Erscheinungen auf; 18,8 % (272/1445) der Expositionen gingen mit leichten/geringen Vergiftungen, 0,6 % (9/1445) mit mittleren/mäßigen Vergiftungen einher. Keine Vergiftung wurde als schwer eingeschätzt; bei 66 der insgesamt 1511 Expositionen war der Schweregrad allerdings nicht klar einschätzbar (in der vorherigen %‑Rechnung nicht berücksichtigt). Es gab keine Todesfälle in dieser Altersgruppe.

Eine ärztliche oder Laienintervention wurde in 91,2 % (1373/1506) der Fälle empfohlen, wobei eine stationäre Behandlung bei 2,9 % (43/1506) bzw. eine ambulante Intervention bei 7,6 % (114/1506) initiiert werden sollte. In 5 Fällen wurde die Interventionsindikation nicht eingeschätzt (in der %‑Rechnung nicht berücksichtigt).

Bei den stationären Behandlungen der Kleinkinder waren folgende Präparate, bezogen auf die Einzeleinnahmen, ursächlich: Babix®-Inhalat N (8/43), Minzöle (6/43), Sanopin® wern (5/43), Prospan® Hustensaft, Goldgeist® forte (je 4/43), Franzbranntwein (3/43), Transpulmin® Erkältungsbalsam für Kinder, „Eukalyptusöl“, „ätherisches Öl“, „Erkältungsbad“ (je 2/43) sowie Bronchipret® Saft TE, Colchysat® Bürger, „Erkältungsbalsam“, Iberogast® und Pulmotin® (je 1/43).

### Babys (< 1 Jahr)

Die Gruppe der Babys wurde aufgrund der potenziellen Vulnerabilität noch einmal gesondert betrachtet: 24 Einzelpräparate mit 374 Expositionen und insgesamt steigendem Anfragetrend (⌀37,4 Fälle pro Jahr, Range: 22–52 Fälle pro Jahr 𝜏 = 0,9111, *p* = 0,003) bzw. 8 relevante Einzelpräparate (*n* ≥ 10 Anfragen im Gesamtstudienzeitraum) mit 328 Expositionen spielen bei dieser Altersgruppe eine Rolle.

Die 5 häufigsten Expositionsanfragen gab es für Babix®-Inhalat N (34,8 %; 130), Fenchel-Kümmel-Öl (17,1 %; 64), Kümmelöl (12,6 %; 47), Minzöl (7,5 %; 28) und Sanopin® wern (5,1 %; 19). Die Expositionsursachen beruhen zum größten Teil auf Präparateverwechslungen (67,1 %; 251) oder waren akzidentell (27,3 %; 102 – exklusive Verpackungsverwechslungen); seltener war die Ursache ein unsachgemäßer Gebrauch (2,9 %; 11).

82,9 % (290/350) aller Expositionen blieben symptomlos; bei 17,1 % (60/350) der Expositionen konnten leichte/geringe Vergiftungen festgestellt werden. Es gab keine als mittel/mäßig oder schwer eingeschätzten Vergiftungen; bei 24 der insgesamt 374 Expositionen war der Schweregrad allerdings nicht klar einschätzbar (in der vorherigen %‑Rechnung nicht berücksichtigt). Es gab keine Todesfälle.

Eine ärztliche oder Laienintervention wurde in 86,5 % (321/371) der Expositionsfälle angeraten, wobei eine stationäre Behandlung bei 5,9 % (22/371) bzw. eine ambulante Intervention bei 10,5 % (39/371) initiiert werden sollte. In 3 Fällen wurde die Interventionsindikation nicht eingeschätzt (in %‑Rechnung nicht berücksichtigt).

Bei den 22 stationären Behandlungsempfehlungen in der Gruppe der Babys waren folgende Präparate, bezogen auf die Einzeleinnahmen, ursächlich: Minzöl (7/22), Babix®-Inhalat N (5/22), Fenchel-Kümmel-Öl (4/22) sowie je einmal (1/22) Sanopin® wern, Goldgeist® forte, Nelkenöl, Eukalyptusöl, Kümmelöl und ein unbestimmtes ätherisches Öl.

## Diskussion

Es zeigt sich, dass ein Großteil der Anfragen von privaten Haushalten stammt. Viele Präparate werden aufgrund ihrer leichten Zugänglichkeit oft ohne entsprechende medizinische Expertise bzw. Aufsicht eingesetzt. Dabei sind Anwendungsfehler mit unerwünschten Nebenwirkungen oder toxischen Reaktionen erwartbar.

Bei den „epidemiologisch relevanten“ Präparaten (≥ 10 Anfragen im Studienzeitraum) verbleiben 36 Produkte, die übersichtlich zusammengefasst und bewertet werden können (Tab. 1). Derartige Synopsen können für Giftnotrufe, Notaufnahmen, Arztpraxen, Apotheken und den Öffentlichen Gesundheitsdienst eine hilfreiche Handreichung im Beratungsfall sein und auch zur Vorbereitung auf Expositionsanfragen sowie als Schulungs- und Informationsmaterialien dienen.

Ein Großteil der GGIZ-Anfragen zu Präparaten mit pflanzlichen Bestandteilen geht auf orale Expositionen (93,4 %) zurück. Viele der relevanten Präparate (z. B. Babix®-Inhalat N, Minzöl, Erkältungsbäder) sind nur als Flüssigkeiten bzw. in Form von Dosiertropfen auf dem Markt. Es ist anzunehmen, dass flüssige Präparate für die extraorale Anwendung in vergleichbarer Verpackungsaufmachung bzw. in Flaschenabfüllung leicht mit Präparaten für den tatsächlichen oralen Gebrauch (z. B. Hustentropfen) verwechselt werden können. Möglicherweise werden aufgrund der flüssigen Darreichungsform auch falsche „Analogieschlüsse“ bezüglich des Applikationsweges gezogen.

**Abb. 5:**
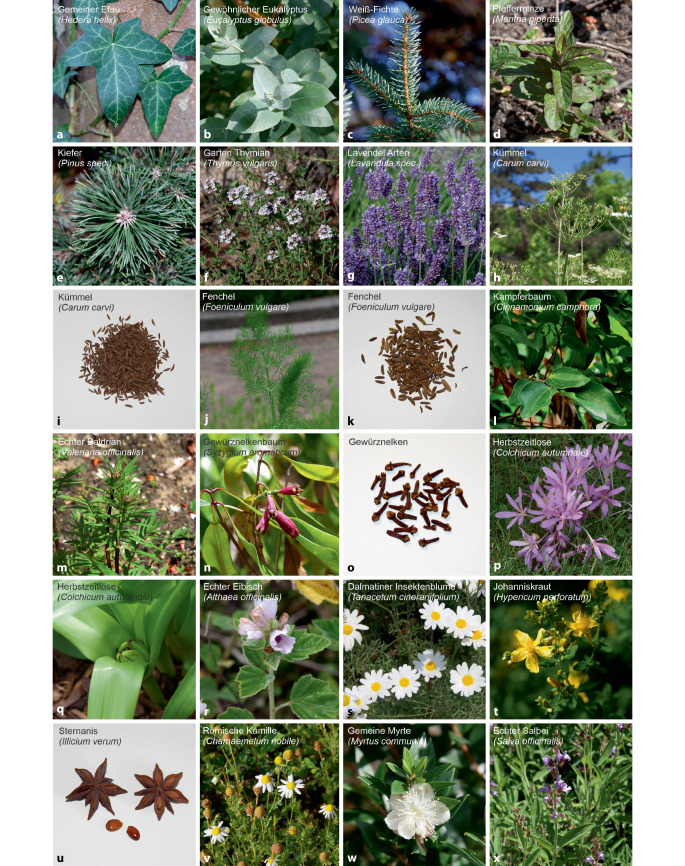
Ausgewählte Heilpflanzen und -bestandteile (Drogen) in den „Zubereitungen mit pflanzlichen Bestandteilen“, die am GGIZ Erfurt im Zeitraum 2013–2022 angefragt wurden

Bei den Präparateklassen waren diverse Zubereitungen mit Extrakten aus Efeublättern (Abb. 5a, z. B. Prospan®-Produkte, Bronchipret® Saft) zu fast einem Viertel bei den Gesamtexpositionen vertreten. Efeublätterextrakte enthalten als relevante pharmakotoxikologische Wirkstoffe u. a. *α*- und *β*-Hederin bzw. Saponine, die im Gemisch antitussiv und bronchospasmolytisch wirken. In höheren Dosen wirken sie jedoch reizend auf den Magen-Darm-Trakt (z. B. Diarrhöen). Die niedrigen modifizierten Litovitz-Risikofaktoren (Tab. 1) zeigen, dass keine schweren und nur wenige mittelschwere Vergiftungen (*n* = 3) bei Efeublätterextraktpräparaten zu erwarten sind. Andererseits ist der allgemeine Informations- und Aufklärungsbedarf in der Bevölkerung ersichtlich hoch.

Neben Hustensaft auf Efeublätterextraktbasis sind relativ häufig Präparate mit Ölen von Eukalyptus und Kiefernnadeln (Abb. 5b, e) vertreten (z. B. Sanopin® wern/Eucabal® Inhalat, Erkältungsbad, Eucabal®-Balsam, Tab. 1). Bei diesen Präparaten, die u. a. längerkettige Kohlenwasserstoffe, Ketone, Terpene, Alkohole und Alkoholderivate aus pflanzlichen Ausgangsstoffen enthalten, liegen die modifizierten Litovitz-Risikofaktoren überwiegend >0, d. h., es kommen mittelschwere bzw. schwere Vergiftungen vor. Bei manchen Präparaten mit ätherischen Ölen und Ethanol (z. B. Franzbranntwein, Klosterfrau® Melissengeist Konzentrat, Pulmotin® Erkältungssalbe, -tropfen, Tab. 1) liegen die modifizierten Litovitz-Risikofaktoren sogar >5, was einem recht hohen realen Intoxikationsrisiko entspricht. Das ausgewiesene Nebenwirkungs- bzw. Intoxikationsspektrum ätherischer Öle ist breit und sollte nicht unterschätzt werden: gastrointestinale Symptome, Bronchokonstriktion, Laryngospasmus, Glottisödem, Nierenversagen, Hautreizungen, Allergien und auch neurologisch-psychiatrische Symptome. Insbesondere bei Patienten mit Asthma bronchiale, Keuchhusten und Pseudokrupp sowie bei Kindern bestehen bei der Anwendung solcher Präparate meist Kontraindikationen bzw. Anwendungsbeschränkungen (z. B. keine Anwendung im Bereich des Gesichtes; Alters- und Dosisbeschränkungen; siehe weiterführend entsprechende Fachinformationen).

Kleinkinder (1 bis <6 Jahre) sind im Datensatz mit 60,2 % der Anfrageraten überrepräsentiert (Abb. 3 und 4), d. h., sie haben auch den größten statistischen Einfluss bei der Gesamtbewertung. Es handelt sich um eine besonders vulnerable Risikogruppe, da die toxischen Dosen z. B. aufgrund kleinerer Verteilungsvolumina und noch geringer organspezifischer und enzymatischer Entgiftungskapazitäten in der Regel niedriger als bei Erwachsenen sind [16, 17]. So wurde auch in den allermeisten Expositionsfällen (> 90 %) bei dieser Altersgruppe eine entsprechende Intervention empfohlen. Die häufigsten Expositionen erfolgten mit kommerziell bekannten Hustensaftpräparaten (Tab. 1).

Bei den Babys (< 1 Jahr) rückt die Bedeutung ätherischer Öle bei den Vergiftungen noch weiter in den Vordergrund als bei allen anderen Altersgruppen, was sich auch in den Altersbeschränkungen der meisten Fachinformationen widerspiegelt (i. d. R. ist die Anwendung bei Kindern <2 Jahre kontraindiziert). Hier war die Ursache der Exposition am häufigsten eine Präparateverwechslung durch die Anwender (z. B. Eltern), wohingegen bei Kleinkindern nachvollziehbar die akzidentelle Exposition am häufigsten auftritt. Unter Präventionsaspekten ist es wünschenswert, dass sich die Verpackungsaufmachungen von Kinder‑/Baby- und Erwachsenenpräparaten optisch möglichst eindeutig und markant voneinander unterscheiden.

Bei den Erwachsenen verschiebt sich das Präparatespektrum hin zu ethanolhaltigen Zubereitungen und pflanzlichen Gichtmitteln. Bei diesen gab es auch am häufigsten schwere Vergiftungsfälle, insbesondere durch Zubereitungen, die den potenten Mitosehemmer Colchicin aus der Herbstzeitlosen (*Colchicum autumnale, *Abb. 5p) enthalten. Das Alkaloid Colchicin weist bei geringer therapeutischer Breite eine hohe und von Körpergewicht, Vorerkrankungen und Arzneimittel-Interaktionen abhängige Toxizität auf [18, 19]; es kann daher schnell zu Nebenwirkungen oder Überdosierungen mit tödlichen Verläufen kommen. Die toxikologische Wirkung hängt aber auch von der Dauer der Exposition, der Konzentration am Wirkort sowie von individuellen Faktoren (z. B. Alter, Metabolismus, Enzympolymorphismen) ab [16]. In dieser Studie hatte ein colchicinhaltiges Präparat mit einem Wert von 31 den höchsten modifizierten Litovitz-Risikofaktor (Tab. 1); es gab auch einen unintendierten Todesfall. Daher bedarf die Behandlung mit colchicinhaltigen Präparaten immer einer intensiven ärztlichen Beratung und Überwachung. Die Vergiftungssymptome bei den Colchicin-Intoxikationen waren allesamt „klassisch“. Mögliche Frühsymptome wie Übelkeit, Erbrechen, Bauchschmerzen oder Diarrhö sollten deshalb stets sehr ernst genommen werden und Anlass sein, unmittelbar ärztliche Hilfe in Anspruch zu nehmen bzw. ein GIZ zu konsultieren (siehe Fachinformationen). Infolge mehrerer berichteter Vergiftungsfälle wurden 2018 bereits die maximalen Packungsgrößen, die Gesamttagesdosen und Fachinformationen/Packungsbeilagen von den Herstellern angepasst [20]; ebenso haben sich die Leitlinien-Empfehlungen zur empfohlenen Tagesdosis inzwischen geändert [21]. Ein Großteil der schweren suizidalen Intoxikationen sind ebenfalls auf colchicinhaltige Produkte zurückzuführen. Das Umfeld von suizidgefährdeten Personen sollte deshalb entsprechend informiert und sensibilisiert sein. Bei Suizidversuchen waren des Weiteren Baldrian-Präparate regelmäßig involviert, wenngleich diese kein reales Vergiftungspotenzial in dieser Studie hatten (modifizierter Litovitz-Risikofaktor: 0).

Bezüglich der schweren Vergiftungen durch alkoholhaltige Zubereitungen sind die hohen Dosen bzw. Volumina, die ingestiert wurden, auffällig, sodass diese eher als vordergründige Ethanolvergiftungen zu werten sind. Alkohole und Alkoholderivate spielen bei der Herstellung (z. B. als Auszugs- bzw. Lösungsmittel), zur Konservierung und als Darreichungsform (z. B. hyperämisierende Tonika) im Sinne von Hilfsstoffen traditionell eine große Rolle. Beispielhaft sei hier Franzbranntwein als Ethanol-Wassergemisch mit den Zusätzen Campher, Thymol und Fichten- bzw. Kiefernnadelöl genannt. Anwendungsbeschränkungen können daher, je nach Applikationsform und Alkoholkonzentration, vielfach bestehen, u. a. aufgrund des Alters (z. B. ab 18 Jahren), bei bestimmten Vorerkrankungen (z. B. Alkoholabhängigkeit, Leberinsuffizienz, Magen-Darm-Geschwüre bei oraler Exposition, Ekzeme und Hautwunden bei dermaler Exposition) sowie bei Schwangeren und Stillenden. Wie auch aus der Literatur bekannt, ist Alkohol bei Suiziden und Suizidversuchen häufig involviert [22]: Klosterfrau® Melissengeist Konzentrat (79 Vol.-% Ethanol) kam im Studienzeitraum mutmaßlich als Ersatz für nicht verfügbare alkoholische Getränke zur Anwendung. In einem letalen Fall wurde dabei ein „Koma mit Azidose“ beschrieben, wie es klassischerweise auch als alkoholische Ketoazidose bei der Intoxikation mit ethanolhaltigen Getränken durch Bildung und Akkumulation von Acetaldehyd, Essigsäure, Lactat und *Beta*-Hydroxybuttersäure im Blut auftritt [23]. Die Behandlung erfolgt analog zur konventionellen Ethanolvergiftung [24, 25]. Hochprozentige Präparate (z. B. Einzelprodukte mit ca. 80 Vol.-% Ethanol in dieser Studie) mit großen Volumina haben daher ein relativ hohes Vergiftungspotenzial, das sich u. a. an den modifizierten Litovitz-Risikofaktoren zeigt (z. B. 8,3 für Klosterfrau® Melissengeist Konzentrat, s. Tab. 1). Das alkoholische Destillat aus 13 Heilkräutern ist in der Literatur auch als Schnüffelstoff („Ersatz-Rauschdroge“) beschrieben [25].

Es empfiehlt sich stets, die Hinweise auf der Verpackung und die Fachinformation zu beachten sowie orale und extraorale Präparate möglichst getrennt voneinander (z. B. in unterschiedlichen Arzneimittelschränken oder Arzneimittelfächern) zu lagern. Auch sollten Zubereitungen mit pflanzlichen Bestandteilen stets außerhalb der Reichweite von Kindern, kognitiv eingeschränkten oder potenziell suizidalen Personen aufbewahrt werden. Sofern Ärzte entsprechende Präparate empfehlen, sollten sie die Anwender grundsätzlich über die korrekte Anwendungsweise aufklären. Gleiches gilt für das Fachpersonal bei der Abgabe in Apotheken. Der Umfang der Aufklärung kann sich auch an der Höhe der modifizierten Litovitz-Risikofaktoren (Tab. 1) orientieren.

Bei bestimmungsgemäßer Anwendung sind unerwünschte Arzneimittelwirkungen bei Zubereitungen mit pflanzlichen Bestandteilen allerdings nur in wenigen Fällen ein Konsultationsgrund, was die hohe Sicherheit dieser Präparate hierzulande unterstreicht. Auch zeigte sich in dieser Studie nur ein sehr geringes Missbrauchspotenzial (z. B. als Psychedelika).

Die vorliegende Studie weist mehrere inhärente Limitationen auf: Zum einen ist die Repräsentativität der Daten eingeschränkt. Aus der Werbung bekannte oder besonders populäre Präparate scheinen in den Anfrageraten überrepräsentiert zu sein, was Verzerrungen zur Folge haben könnte. Zudem fehlen genaue Daten zu Verkaufszahlen sowie zu Anwendungs- beziehungsweise Konsumraten in der Gesamtbevölkerung, sodass aus den absoluten Häufigkeiten keine Rückschlüsse auf die tatsächliche Gefährlichkeit einzelner Präparate gezogen werden können. Weiterhin ist ein gewisser „Anrufer-Bias“ im Sinne eines Nocebo-Effekts denkbar, bei dem gesundheitliche Risiken subjektiv überbewertet werden, ohne dass ein kausaler Zusammenhang besteht. Andererseits wenden sich Betroffene mit ausgeprägten Symptomen eher an ein Giftinformationszentrum (GIZ), was die Daten zusätzlich beeinflusst. Die Grundgesamtheit („Nenner-Wert“) der jeweiligen Einzeleinnahmen kann aus den GIZ-Daten naturgemäß nicht ermittelt werden. Auch sind die Nachverfolgungs- und Verifikationsmöglichkeiten sowie die Validität des PSS im telefonischen Setting begrenzt. Die Symptomschwere beschreibt daher nur den maximalen am Telefon erhobenen Schweregrad – meist ohne proaktive Verlaufsbeobachtungen. Insbesondere bei unspezifischen Beschwerden bleibt die kausale Zuordnung mit einer Unsicherheit behaftet. Bei der Kausalitätsbeurteilung von Einzelsubstanzen sollte zudem berücksichtigt werden, dass es sich bei vielen pflanzlichen Heilpräparaten um komplexe Stoffgemische handelt.

## Fazit

Die Analyse von Expositionsanfragen zu Zubereitungen mit pflanzlichen Bestandteilen am Gemeinsamen Giftinformationszentrum Erfurt zeigt, dass entsprechende Präparate häufig Anlass für Beratungsanfragen sind, insbesondere bei Kleinkindern. Die meisten Expositionen erfolgen im häuslichen Umfeld und beruhen auf akzidentellen Einnahmen oder Präparateverwechslungen; klinisch verlaufen sie überwiegend symptomlos oder mit leichten Beschwerden. Einzelne Zubereitungen – insbesondere colchicinhaltige Arzneimittel oder ethanolhaltige Präparate – können jedoch mit einem relevanten Intoxikationsrisiko bis hin zu letalen Verläufen verbunden sein. Daraus ergibt sich die Bedeutung präventiver Maßnahmen, etwa einer sicheren Aufbewahrung, einer besseren Differenzierung von Verpackungen sowie einer Aufklärung durch Fachpersonal.

Die Aussagekraft der Ergebnisse ist begrenzt, da Beratungsanfragen die Datengrundlage bilden, bei denen weder eine vollständige Erfassung aller Variablen noch eine direkte Abschätzung populationsbezogener Risiken möglich ist. Zukünftige Studien sollten daher populationsbasierte Daten zu Anwendungshäufigkeiten sowie prospektive toxikologische Verlaufsbeobachtungen einbeziehen. Insgesamt verdeutlichen die Ergebnisse, dass auch pflanzliche Zubereitungen ein relevantes toxikologisches Potenzial besitzen und deshalb in Prävention, Beratung und klinischer Bewertung angemessen berücksichtigt werden sollten.

## Supplementary Information

ESM1: Zusatzmaterial 1

## Data Availability

Die während der vorliegenden Studie erzeugten und/oder analysierten Datensätze sind auf begründete Anfrage bei der Korrespondenzperson erhältlich.

## References

[1] Gemeinsame Giftinformationszentrum Erfurt (2025) Anfragen zu Arzneimitteln. https://www.ggiz-erfurt.de/giftinformation.html. Zugegriffen: 12. Dezember 2025

[2] Zhang J, Onakpoya IJ, Posadzki P, Eddouks M (2015) The safety of herbal medicine: from prejudice to evidence. Evid Based Complement Alternat Med 2015:316706. 10.1155/2015/316706 25838831 10.1155/2015/316706 PMC4370194

[3] Ekor M (2014) The growing use of herbal medicines: issues relating to adverse reactions and challenges in monitoring safety. Front Pharmacol 4:177. 10.3389/fphar.2013.00177 24454289 10.3389/fphar.2013.00177 PMC3887317

[4] Bundesverband der Pharmazeutischen Industrie e. V. (2023) Markt für Phytopharmaka in Deutschland 2019-2022. OTC-Sonderpublikation. https://www.bpi.de/index.php?eID=dumpFile&t=f&f=77593&token=f5f580db680c71354fe00b5362a158e6b791227d. Zugegriffen: 30. Mai 2025

[5] Posadzki P, Watson LK, Ernst E (2013) Adverse effects of herbal medicines: an overview of systematic reviews. Clin Med. Lond, Bd 13, S 7–12 10.7861/clinmedicine.13-1-7 10.7861/clinmedicine.13-1-7 PMC5873713 23472485

[6] Efferth T, Kaina B (2011) Toxicities by herbal medicines with emphasis to traditional Chinese medicine. Curr Drug Metab 12:989–996. 10.2174/138920011798062328 21892916 10.2174/138920011798062328

[7] Byard RW (2010) A review of the potential forensic significance of traditional herbal medicines. J Forensic Sci 55:89–92. 10.1111/j.1556-4029.2009.01252.x 20412155 10.1111/j.1556-4029.2009.01252.x

[8] Marcus DM, Grollman AP (2016) Toxicity of Botanical Medicines: An Overlooked Global Health Problem. Am J Public Health 106:16–17. 10.2105/AJPH.2015.302937 26562106 10.2105/AJPH.2015.302937 PMC4695924

[9] Valdivia-Correa B, Gómez-Gutiérrez C, Uribe M, Méndez-Sánchez N (2016) Herbal Medicine in Mexico: A Cause of Hepatotoxicity. A Critical Review. Int J Mol Sci 17:235. 10.3390/ijms17020235 10.3390/ijms17020235 PMC4783966 26891292

[10] Charen E, Harbord N (2020) Toxicity of Herbs, Vitamins, and Supplements. Adv Chronic Kidney Dis 27:67–71. 10.1053/j.ackd.2019.08.003 32147004 10.1053/j.ackd.2019.08.003

[11] Bazzano AN, Var C, Grossman F, Oberhelman RA (2017) Use of Camphor and Essential Oil Balms for Infants in Cambodia. J Trop Pediatr 63:65–69. 10.1093/tropej/fmw013 27370817 10.1093/tropej/fmw013 PMC5301968

[12] Mathew T, Kamath V, Kumar RS et al (2017) Eucalyptus oil inhalation-induced seizure: A novel, underrecognized, preventable cause of acute symptomatic seizure. Epilepsia Open 2:350–354. 10.1002/epi4.12065 29588965 10.1002/epi4.12065 PMC5862119

[13] Finkelstein Y, Aks SE, Hutson JR et al (2010) Colchicine poisoning: the dark side of an ancient drug. Clin Toxicol (phila) 48:407–414. 10.3109/15563650.2010.495348 20586571 10.3109/15563650.2010.495348

[14] Wendt S, Linden D, Prasa D, Franke H (2025) The Frequency of Inquiries About Suspected Poisoning With Botanical Drugs. Dtsch Ärztebl Int 122:562–563. 10.3238/arztebl.m2025.0106 41118564 10.3238/arztebl.m2025.0106 PMC12620898

[15] Persson HE, Sjöberg GK, Haines JA, Pronczuk de Garbino J (1998) Poisoning severity score. Grading of acute poisoning. J Toxicol Clin Toxicol 36:205–213. 10.3109/15563659809028940 9656975 10.3109/15563659809028940

[16] Wendt S, Lübbert C, Begemann K, Prasa D, Franke H (2022) Poisoning by plants. Dtsch Ärztebl Int 119:317–324. 10.3238/arztebl.m2022.0124 35140011 10.3238/arztebl.m2022.0124 PMC9453220

[17] Wendt S, Prasa D, Lübbert C, Begemann K, Franke H (2023) Expositionen mit Fruchtpflanzen in Deutschland im Zeitraum 2010–2019 : Auswertung der Datenbank des Gemeinsamen Giftinformationszentrums Erfurt (GGIZ) (Exposures to fruit plants in Germany from 2010–2019 : Analysis of the Erfurt joint poison information center database). Bundesgesundheitsblatt Gesundheitsforschung Gesundheitsschutz 66:1423–1433. 10.1007/s00103-023-03780-7 10.1007/s00103-023-03780-7 PMC10667429 37828294

[18] Slobodnick A, Shah B, Pillinger MH, Krasnokutsky S (2015) Colchicine: old and new. Am J Med 128:461–470. 10.1016/j.amjmed.2014.12.010 25554368 10.1016/j.amjmed.2014.12.010 PMC4684410

[19] Boyadzhieva Z, Ruffer N, Krusche M (2021) Colchicin: altes Medikament mit neuem Nutzen : Einsatz in der Rheumatologie und darüber hinaus (Colchicine: old medication with new benefits : Use in rheumatology and beyond). Z Rheumatol 80:647–657. 10.1007/s00393-021-01017-z 10.1007/s00393-021-01017-z PMC8181537 34097101

[20] Bundesinstitut für Arzneimittel und Medizinprodukte (2018) Risikoinformation für Colchicum-Dispert® und Colchysat®Bürger der Firma Bürger. Wirkstoff: Colchicin. https://www.bfarm.de/SharedDocs/Risikoinformationen/Pharmakovigilanz/DE/RI/2018/RI-colchicin.html. Zugegriffen: 9. Dezember 2024

[21] Deutsche Gesellschaft für Rheumatologie und Klinische Immunologie e. V. (2024) Diagnostik und Therapie der Gicht. Version 2024. https://register.awmf.org/de/leitlinien/detail/060-005. Zugegriffen: 9. Dezember 2024

[22] Norström T, Rossow I (2016) Alcohol Consumption as a Risk Factor for Suicidal Behavior: A Systematic Review of Associations at the Individual and at the Population Level. Arch Suicide Res 20:489–506. 10.1080/13811118.2016.1158678 26953621 10.1080/13811118.2016.1158678

[23] Long B, Lentz S, Gottlieb M (2021) Alcoholic Ketoacidosis: Etiologies, Evaluation, and Management. J Emerg Med 61:658–665. 10.1016/j.jemermed.2021.09.007 34711442 10.1016/j.jemermed.2021.09.007

[24] Mirijello A, Sestito L, Antonelli M, Gasbarrini A, Addolorato G (2023) Identification and management of acute alcohol intoxication. Eur J Intern Med 108:1–8. 10.1016/j.ejim.2022.08.013 35985955 10.1016/j.ejim.2022.08.013

[25] Ludewig R, Regenthal R (2015) Akute Vergiftungen und Arzneimittelüberdosierungen. Schnell- und Hintergrundinformationen zu Erkennung, Verlauf, Behandlung und. Verhütung, Bd 11. völlig neu bearbeitete Auflage. Wissenschaftliche Verlagsgesellschaft Stuttgart, Stuttgart

