# Supplementary material for: Exposure inquiries related to preparations containing plant-based ingredients in Germany: analysis of inquiries (2013–2022) at the Joint Poison Information Center in Erfurt
Source: Bundesgesundheitsblatt Gesundheitsforschung Gesundheitsschutz. 2026 Apr 29;69(6):644–56. [Article in German] doi: 10.1007/s00103-026-04237-3 (PMC13212780; doi:10.1007/s00103-026-04237-3)
Supplement: Supplementary file 1 — ESM1: Zusatzmaterial 1 [file 103_2026_4237_MOESM1_ESM.pdf]

Onlinematerial

Tab. Z1: Modifizierter Poisoning Severity Score (PSS) nach PERSSON et al. zur Beurteilung der Vergiftungsschwere. Tabelle modifiziert nach Persson et al. (1998).

**Organsystem**	**Keine Symptome**	**Leichte Symptome**	**Mittelschwere Symptome**	**Schwere Symptome**	**Tod**
	Keine Symptome oder Anzeichen im Zusammenhang mit einer Vergiftung durch Heilpflanzenpräparate	*Leichte, vorübergehende und spontan zurückgehende Symptome im Zusammenhang mit einer Vergiftung durch Heilpflanzenpräparate*	*Ausgeprägte oder anhaltende Symptome im Zusammenhang mit einer Vergiftung durch Heilpflanzenpräparate*	*Schwere oder lebensbedrohliche Symptome im Zusammenhang mit einer Vergiftung durch Heilpflanzenpräparate*	*Todesfolge im Zusammenhang mit einer Vergiftung durch Heilpflanzenpräparate*
	**0**	**1**	**2**	**3**	**4**
**Gastrointestinaltrakt**	Symptomlosigkeit	• Bauchschmerz, Erbrechen, Durchfall,<br>• Schleimhautreizung (1° Verätzung), Aphthen im Mund<br>• endoskopisch: Schleimhautrötung Schwellung	• Erbrechen Bauchschmerzen, Durchfall, länger als 2 Stunden anhaltend<br>• 2° oder 3° Verätzung in kleinem Schleimhautbereich<br>• leichte Schluckstörung<br>• endoskopisch: Ulkus	• starkes Bluterbrechen, Perforation<br>• 2° oder 3° Verätzung in größerem Schleimhautbereich<br>• schwere Schluckstörung<br>• endoskopisch: tiefes Ulkus, zirkumferente Läsion, Perforation	Tod
**Respirationstrakt**		• Reizgefühl, Husten, Kurzatmigkeit, leichte Atemnot, gering gradiger Bronchospasmus<br>• auffälliger Röntgen-Thorax-Befund ohne Beschwerden	• anhaltender Husten, Bronchospasmus, Atemnot, Stridor<br>• reduzierte Sauerstoffsättigung<br>• auffälliger Röntgen-Thorax-Befund mit leichten/mäßigen Beschwerden	• manifeste Ateminsuffizienz (z. B. schwerer Bronchospasmus, Atemwegsverlegung, Glottisödem, Lungenödem, Atemnotsyndrom (ARDS), Pneumonitis, Pneumothorax)<br>• auffälliger Röntgen- Thorax-Befund mit starken Beschwerden	
**Nervensystem**		• Benommenheit, Schwindel, Ohrgeräusch, innere Unruhe<br>• Ataxie, leichte extrapyramidal- motorische Bewegungsstörungen<br>• leichte cholinerge oder anticholingere Symptome<br>• Missempfinden<br>• leichte Seh- oder Hörstörungen	• Bewusstlosigkeit mit gezielter Reaktion auf Schmerzreize<br>• kurzer Atemstillstand verlangsamte Atmung<br>• Verwirrtheit, Agitiertheit, Halluzination, Delir<br>• Krampfanfälle (lokal oder generalisiert)<br>• starke extrapyramidal-motorische Bewegungsstörungen	• Bewusstlosigkeit ohne oder mit ungezielter Reaktion auf Schmerzreize<br>• Atemantriebsstörung<br>• starke Agitiertheit<br>• häufige Krampfanfälle oder *Status epilepticus*, Opisthotonus<br>• generalisierte oder lebensbedrohliche Muskellähmung<br>• Erblindung oder Taubheit	

			• starke cholinerge oder anticholingere Symptome<br>• umschriebene, nicht lebensbedrohliche Muskellähmung<br>• starke Seh- oder Hörstörungen		
**Herz-Kreislauf-System**		• isolierte Extrasystolen<br>• leichter oder kurzzeitiger Blutdruckanstieg oder Blutdruckabfall	• mäßige Sinusbradykardie (Erw.: 40-50/min, Kinder: 60-80/min, Neugeborene: 80-90/min)<br>• mäßige Sinustachykardie (Erw.: 140-180/min, Kinder: 160-190/min, Neugeborene: 160-200/min)<br>• häufige Extrasystolen, Vorhofflattern oder -flimmern<br>• AV-Block 1-2°<br>• verbreiterter QRS-Komplex oder verlängerte QT-Zeit/Repolarisationsstörungen<br>• Myokardischämie<br>• starker Blutdruckanstieg oder Blutdruckabfall	• schwere Sinusbradykardie (Erw.: <40/min, Kinder: <60/min, Neugeborene: <80/min)<br>• starke Sinustachykardie (Erw.: >180/min, Kinder: >190/min, Neugeborene: >200/min)<br>• lebensbedrohliche Herzrhythmusstörungen,<br>• AV-Block 3°<br>• Herzstillstand<br>• Myokardinfarkt<br>• Kreislaufschock<br>• hypertensive Krise	
**Niere**		• minimale Proteinurie/ Hämaturie	• massive Proteinurie/Hämaturie<br>• Nierenfunktionsstörung (z.B. Oligurie, Polyurie Serumkreatinin von ~200-500 µmol/L)	• Nierenversagen (z.B. Anurie, Serumkreatinin von >500 µmol/L)	
**Leber**		• minimaler Anstieg der Transaminasen (ASAT, ALAT ~2-5 x normal)	• deutlicher Anstieg der Transaminasen (ASAT, ALAT ~5-50 x normal)<br>• keine weiteren auffälligen biochemischen Marker (z. B. Ammoniak, Gerinnungsfaktoren)<br>• keine klinischen Hinweise auf Leberfunktionsstörung	• extremer Anstieg der Transaminasen (>50 x normal)<br>• weitere pathologische biochemische Marker (z. B. Ammoniak, Gerinnungsfaktoren)<br>• klinische Hinweise auf Leberversagen	
**Muskulatur**		• milder Schmerz, Spannung<br>• CPK ~250-1.500 IU/L	• mäßige Schmerzen, Steifheit, Krämpfe, Faszikulationen<br>• Rhabdomyolyse<br>• CPK ~1.500-10.000 IU/L	• starke Schmerzen, extreme Steifheit, generalisierte Krämpfe und Faszikulationen<br>• Rhabdomyolyse mit Komplikationen<br>• CPK >10.000 IU/L<br>• Kompartmentsyndrom	

**Metabolismus**		• leichte Säure-Base-Störungen<br>(HCO_3^- ~15-20 oder 30-40 mmol/L<br>pH ~7,25-7,32 oder 7,50-7,59)<br>• milde Elektrolyt- u. Flüssigkeitsstörungen (K^+ 3,0-3,4 oder 5,2-5,9 mmol/L)<br>• leichte Hypoglykämie (~50-70 mg/dL<br>oder 2,8-3,9 mmol/L bei Erwachsenen)<br>• Hyperthermie von kurzer Dauer	• stärker ausgeprägte Säure-Base-Störungen (HCO_3^- ~10-14 oder >40 mmol/L; pH ~7,15-7,24 oder 7,60-7,69)<br>• stärker ausgeprägte Elektrolyt- u. Flüssigkeitsstörungen (K^+ 2,5-2,9 oder 6,0-6,9 mmol/L)<br>• stärkere Hypoglykämie (~30-50 mg/dL oder 1,7-2,8 mmol/L bei Erwachsenen)<br>• Hyperthermie von längerer Dauer	• schwere Säure-Base-Störungen (HCO_3^- <10 mmol/L; pH <7,15 oder >7,7)<br>• schwere Elektrolyt- und Flüssigkeitsstörungen (K^+ <2,5 oder >7,0 mmol/L)<br>• schwere Hypoglykämie (<30 mg/dL oder <1,7 mmol/L bei Erwachsenen)<br>• gefährliche Hypo- oder Hyperthermie	
**Blutsystem**		• leichte Hämolyse<br>• leichte Methämoglobinämie (MetHb ~10-30%)	• mäßige Hämolyse<br>• ausgeprägtere Methämoglobinämie (MetHb ~30-50%)<br>• Gerinnungsstörungen ohne Blutung<br>• mäßige Anämie, Leukopenie, Thrombozytopenie	• massive Hämolyse<br>• schwere Methämoglobinämie (MetHb >50%)<br>• Gerinnungsstörungen mit Blutungen<br>• schwere Anämie, Leukopenie, Thrombozytopenie	
